# Personality distribution of Canadian medical students: A first look

**Published:** 2018-05-31

**Authors:** June Harris, Donald McKay

**Affiliations:** 1 Faculty of Medicine, Memorial University of Newfoundland, Newfoundland, Canada

## Abstract

**Background:**

Personality is one of the key elements in professional identity formation and is self-identified as one of the top two influences for Canadian medical graduates when making a specialty choice yet little is known about the personalities of Canadian medical students. This study is the first to report personality data regarding Canadian medical students.

**Methods:**

Personality is one of the key elements in professional identity formation and is self-identified as one of the top two influences for Canadian medical graduates when making a specialty choice yet little is known about the personalities of Canadian medical students. This study is the first to report personality data regarding Canadian medical students.

**Results:**

The data were analyzed using Chi square. The distribution of personalities [Guardian, Idealist, Artisan, Rational] for medical students differs from the distribution reported for the general Canadian population. The distribution of personalities is similar for each Canadian medical school.

**Conclusion:**

Results from this first national accounting of the personalities of Canadian medical students suggest either that the personalities of medical school applicants differ from the general population or that personality affects medical school admissions success. Knowing the personalities of medical students could be important for medical schools in such areas as admissions, career counselling and professional identity formation.

## Introduction

In the absence of any published national data on the personality distribution of Canadian medical students, we set out to collect preliminary information from medical students in schools across Canada and compare this with data for the general Canadian population.

The complete absence of published information on the personalities of Canadian medical school students is remarkable when one considers the many published studies that show how personality influences the interaction of medical students with the medical education system throughout its continuum.

Personality type may influence admissions decisions. Admissions committee members set criteria that influence the selection of a certain personality type or tend to select students who are like themselves.^1,2^ Some speculate that a personality measure may be useful as part of the admissions process in an effort to detect possible unprofessional behaviour.^3^ A model has been proposed for “ethically defensible selection decisions” that includes personality along with its other components of self-selection, academic achievement, cognitive ability, and interview/task behavioural scores.^4^ Additionally, the potential link between specific personality traits and clinical performance may warrant inclusion of this predictor in the selection process.^5^

Personality and socialization are two major components of professional identity formation.^6^ Several studies have reported how socialization shapes the professional identity of medical students,^7-9^ yet we could find no similar studies related to personality. Students, however, bring different attitudes and behaviours to the profession, and students themselves suggest that these factors need to be understood and considered when integrating professional identity activities into a medical education program^10^ – activities such as role-modelling^11^ or career counselling.^12-14^ A commentary in Student BMJ described how the Keirsey Temperament Sorter (KTS) can give insight into the importance of knowing your own, as well as colleagues’ personalities, when choosing a medical specialty and outlines that knowing about personality can improve interpersonal relations.^15^

Various personality assessment inventories have gained popularity within the past several years (e.g., Big Five, NEO-4). Over the past 20 years, however, a few personality studies of medical and other health professions have been based on the KTS.^16-18^ We used the KTS-II for our study as it was the only online personality assessment tool available at the outset of this study. Even though the name of the instrument contains the term temperament, we use the synonymous term, personality, in order to provide uniformity throughout this paper.

The manner by which people communicate, gather information, make decisions and organize themselves has been characterized by the KTS-II in four pairs of preferences that describe different personality traits or characters:

- communication style – Extrovert/Introvert [E/I]
- information-gathering style – Sensing/iNtuitive [S/N]
- decision-making style – Thinking/Feeling [T/F]
- management style – Judging/Perceiving [J/P]

The answers to the KTS-II 70-item questionnaire enable sorting of these dichotomous pairs so that a person’s personality can be determined by totalling the answers for each choice; the higher number is the preference. The KTS-II combines these preferences to express personality partially in terms of one’s nature and a person is categorized into only one personality:^19,20^

- Guardian [SJ] – security seeking
- Artisan [SP] – stimulation seeking
- Idealist [NF] – human potential or identity seeking
- Rational [NT] – knowledge seeking

We surveyed the Canadian medical student population over a 10-year period to determine the answer to the following central research question: Does the personality distribution of the Canadian medical school student population differ from the general Canadian population? As a sub-question, we also wanted to answer: Is the distribution of personalities the same for each school in Canada?

## Methods

### Setting and study design

Administrators at each of Canada’s existing 17 medical schools were sent an invitation requesting the participation of their students in this study. If there was no response, they were contacted in subsequent years until the study was closed. Two schools (i.e., Memorial University of Newfoundland (MUN) and McGill University (McGill)) joined the study in its first year. With the exception of MUN, when schools joined the study, all current students were eligible to participate. At the inception of the study, one graduating class of students at MUN had completed the KTS-II as part of an annual workshop and were not included in the study. Ultimately, nine classes (i.e., group of students in a particular graduation year) from McGill and The University of Western Ontario (Western), eight classes from MUN, Dalhousie University (Dalhousie), and University of Toronto (Toronto), six classes from Université Laval (Laval), five classes from Université de Sherbrooke (Sherbrooke) and University of Alberta (Alberta), four classes from University of British Columbia (UBC), and three classes from University of Manitoba (Manitoba) were eligible to participate. We used a descriptive study design. This study was approved by the Human Investigations Committee at Memorial University of Newfoundland. Where required, participating schools completed internal review board applications.

### Study protocol

The KTS-II was available online so it was accessible to all medical students.^20^ With one exception,^*^ administrators at participating medicals schools sent the names of all enrolled medical students to the principal investigator. A unique access code for each student was developed by the principal investigator and these codes were sent to Keirsey.com for entry into a database. The participating school supplied the year of program entry and gender for each student in all four years. The principal investigator annually notified all students (via email to each school’s administrative staff person) that the survey was available and volunteers were needed. Each volunteer received a unique access code to a webpage and completed the online KTS-II. Each student and the principal investigator received an individualized report of the personality assessment. Students were eligible to complete the survey only once and could complete it at any time during their undergraduate medical training.

### Sample size and sampling methods

A convenience sampling method was used within the ten participating medical schools. Students self-selected to volunteer for the study. All medical students enrolled at a participating school were invited via email. Data from Sherbrooke (n = 48) were excluded^t^ from all analyses as the method of student recruitment differed at that school. Using the sample size calculator from Creative Research Systems online^21^ the sample size needed is 3422 for a confidence level of 95%.

### Outcome measures

The percentages of personalities were compared among nine participating Canadian medical schools and between Canadian medical students and the general Canadian population using data provided by the Keirsey Team.^22^

### Data analysis

All the data are of the categorical type and were analyzed using SPSS, v.22.^23^ The variables are nominal so not expected to follow a normal distribution; therefore, analyses of frequencies were done using non-parametric Chi-square tests. Tests of significance for schools were determined iteratively meaning that one or more schools were removed and a recalculation done to test the significance of those remaining.

## Results

A total of ten out of the 17 Canadian medical school administrators accepted the invitation to participate accessing the majority of provinces with medical schools and, as described in Methods, data from one school were excluded. Over the course of the study, 8806 students from the remaining nine medical schools were eligible to participate. A response rate of 39% (n = 3435) was achieved.

Women comprised 56% of the overall eligible student population and accounted for 63% of the volunteer participants in this study. The response rate for men was 32% and for women was 45%.

Participants could complete the survey only once and were permitted to complete it in any one of the 4 years of medical school. The largest number of students completed it in first year [Year 1: n=1643, Year 2: n=783; Year 3: n=484; Year 4: n=525] (see Table 1). The distribution of personalities showed that the majority of medical students in every year were Guardians and Idealists [Year 1 – 83%, Year 2 – 83%, Year 3 – 83%, Year 4 – 84%] (see Table 2).

**Table 1:** Demographics of each participating Canadian medical school

	Data for Grad Classes of:	Year 1 Participants N (%)	Year 2 Participants N (%)	Year 3 Participants N (%)	≥ Year 4 Participants #N (%)	Total Participants N (%)	Total Eligible N	Response Rate %
**Alberta**	2008-2012	120 (47)	62 (24)	26 (10)	46 (18)	254 (7)	695	37
**Dalhousie**	2005-2012	199 (58)	92 (27)	26 (8)	24 (7)	341 (10)	744	46
**Laval**	2007-2012	121 (35)	57 (16)	46 (13)	125 (36)	349 (10)	1217	29
**Manitoba**	2010-2012	0 (0)	25 (31)	22 (28)	33 (41)	80 (2)	319	25
**McGill**	2004-2012	349 (48)	198 (27)	133 (18)	49 (7)	729 (21)	1488	49
**MUN**	2005-2012	298 (86)	38 (11)	3 (1)	8 (2)	347 (10)	501	69
**Toronto**	2005-2012	218 (36)	137 (23)	119 (20)	132 (22)	606 (18)	1674	36
**UBC**	2009-2012	55 (21)	89 (34)	59 (22)	62 (23)	265 (8)	974	27
**Western**	2004-2012	283 (61)	85 (18)	50 (11)	46 (10)	464 (14)	1194	39
**Total**		1643 (48)	783 (23)	484 (14)	525 (15)	3435 (100)	8806	

**Table 2:** Distribution of personalities in each year of medical school

	Year 1 N (%)	Year 2 N (%)	Year 3 N (%)	≥Year 4 N (%)
**Guardian (SJ)**	905 (55)	448 (57)	279 (58)	320 (61)
**Idealist (NF)**	453 (28)	202 (26)	121 (25)	122 (23)
**Artisan (SP)**	165 (10)	94 (12)	63 (13)	62 (12)
**Rational (NT)**	120 (7)	39 (5)	21 (4)	21 (4)

**Figure 1:**
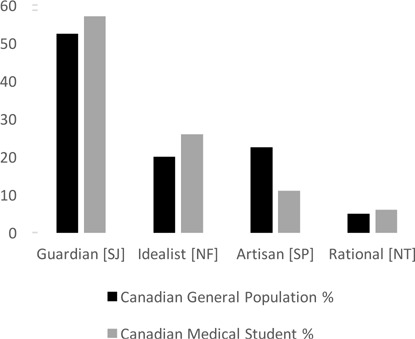
Distribution^†^ of personalities in the Canadian general population^‡^ compared to Canadian medical students

The distribution of the four Keirsey personalities for the Canadian medical students in this study was 57% Guardians [SJ], 26% Idealists [NF], 11% Artisans [SP], and 6% Rationals [NT]. When each school was examined on an individual basis, the distributions of personalities were similar to those of the weighted average such that the majority of medical students at every participating Canadian medical school were Guardians (see Table 3). All schools were included in the first analysis giving X^2^=48.57, df=24, p<0.01; Cramer’s V=0.069, p<0.002. With both Laval and UBC removed for the second analysis, X^2^=16.53, df=18, p>0.05; Cramer’s V=0.044, p<0.556. Although the percent distributions of the four personalities at Laval and UBC significantly differed from the rest of the seven schools, all schools were found to have a similar frequency distribution for the four personalities (Guardian>Idealist>Artisan>Rational). Schools with high response rates showed the same frequency distribution as those with low response rates. The distribution of personalities observed in Canadian medical students differed significantly from that of the general Canadian population [X^2^=276.36, df=3, p<0.000; Cramer’s V=0.008, p< 0.000] (see Figure 1).

**Table 3:** Percent distribution of personalities in each participating Canadian medical school

	Guardian [SJ] N (%)	Idealist [NF] N (%)	Artisan [SP] N (%)	Rational [NT] N (%)
**Alberta**	144 (57)	64 (25)	28 (11)	18 (7)
**Dalhousie**	187 (55)	96 (28)	41 (12)	17 (5)
**Laval**^†^	212 (61)	66 (19)	56 (16)	15 (4)
**Manitoba**	40 (50)	25 (31)	10 (13)	5 (6)
**McGill**	395 (54)	202 (28)	78 (11)	54 (7)
**MUN**	208 (60)	91 (26)	36 (10)	12 (4)
**Toronto**	353 (58)	162 (27)	55 (9)	36 (6)
**UBC**^†^	167 (63)	52 (20)	36 (14)	10 (4)
**Western**	246 (53)	140 (30)	44 (9)	34 (7)
**Average**^‡^	**1952 (57)**	**898 (26)**	**384 (11)**	**201 (6)**

† Using Chi square, significantly different from other schools overall

‡ ‘Weighted average’ percentages rounded to nearest whole number

## Discussion

Whether the personality traits of entering medical students have anything to do with the kinds of doctors being educated may be an interesting academic question, but for those medical students accepted into our programs, a better understanding of personalities within our medical schools and by students themselves may impact upon the creation of a professional identity that will assist them select a successful career path. The data in this study provide a foundation in understanding how the distribution of personalities in Canadian medical students might be used to facilitate the formation of professional identities and strategically guide students in career planning activities. These data may also be important in admissions and help guide human resource planning issues among specialty choices that are poorly subscribed by Canadian medical graduates.

The results of this study were gathered over a 10-year sampling window with data from 3,435 medical students from nine of Canada’s 17 medical schools. They showed that the distribution of personalities was similar among the medical schools analyzed and that the distribution is different from that of the general Canadian public. We acknowledge that even though the Chi-square is significant for the latter, the Cramer V of .008 indicates that there is a low strength of association so there may be other variables contributing to the relationship.

Despite the study strengths, some limitations temper the application of its conclusions. In specific, medical students rather than medical school applicants were assessed. The convenience sampling method used resulted in a 39% overall participation rate and a greater percentage of women participants. A response bias related to the outcome of personality is another possible limitation of this study. This may have been a factor in those schools with a low response rate; perhaps a higher response rate would give a different distribution. Furthermore, we used a personality assessment tool that was easily accessible to students at the study outset and, in the interim, other personality indices became available.

Keirsey proposes that different personalities are drawn to different careers: Guardians want to “contribute to society, and have a sense of security and confidence in their abilities,” Artisans want “a life of action and freedom,” Idealists want “a life of meaning and to help themselves and others grow to be the best that they can be,” and Rationals want to “increase their knowledge base and be highly competent.”^20^ Small-scale studies have been conducted in the health care professions, specifically nursing in Canada, dentistry in the UK, and pediatrics in the US, using the KTS. In nursing, Guardians (52%) and Idealists (19%) formed the majority; in addition, Guardians predominated in all areas of responsibility but Rationals were more prevalent in research/administration and Idealists more prevalent in direct care/teaching.^16^ In dentistry, Guardians (56%) and Idealists (27%) accounted for the majority of dental school applicants.^17^ In a small group of US pediatric residents, a similar distribution to ours was also reported with Guardians (60.7%) and Idealists (26.8%) in the majority and the distribution was significantly different from the general US population.^18^

Among medical schools in Australia, the distribution of personality traits differs from school to school.^24^ In contrast, our data suggest that distributions of medical student personalities across all Canadian medical schools are similar. These differences could be explained by different societal and role-related pressures that are known to mould the identities of students that may vary between Canada and Australia.^6^ Alternatively, differences in the findings may be the result of the personality index used (NEO vs KTS-II) or it may be that the admissions criteria and desired educational outcomes among Australian medical schools may sufficiently differ, so that the personalities of those accepted are likely to reflect this diversity.^24^ Whether the differences for Laval and UBC can be explained by cultural or admissions requirements is a matter for further study.

With respect to personalities, the majority of medical students at every participating Canadian school are Guardians [SJ]. Although Guardian is the most common personality in the Canadian general population, the apparent higher percentage found in medical school may suggest that Guardians are more attracted to the medical profession. Idealists [NF] rank second in the medical student population but rank third in the general Canadian population. Idealists may also be more attracted to the medical profession. The unexpected outcome is the apparent low percentage of Artisans [SP] compared to the general Canadian population. This finding may suggest that either Artisans are not as attracted to the medical profession as other types or, if they are, they are not successful in being admitted into medical school in Canada. In this study, the personality distribution of the applicant pool is unknown, and therefore, the attractiveness of medical school related to personality is unknown. Factors such as admissions criteria or personality traits of interviewers may affect the personality distribution observed among medical students.^1,2^

The recognition of the distribution of personalities among Canadian medical students may be required to develop appropriate approaches to professional identity formation and future studies may build on the results of this study. To illustrate, our data show that the majority (83%) of Canadian medical students are of two common types (by our testing, Guardians and Idealists). People prefer to associate with those who can support and foster them,^25^ and role-modelling plays a part in professional identity formation.^11^ As Rational and Artisan personalities are in the minority of medical students and, by extrapolation, in the minority among physicians, they may have fewer opportunities to observe physician role models who share their same values so they may be at a disadvantage in the development of their professional identities.

Although some question the use of personality assessments in career choice, others give evidence to support their usefulness.^26,27^ Studies continue to show that personality is related to a medical specialty choice^28^ and some are now probing whether the development of professional identity influences specialty choice.^12-14^

The Canadian Graduation Questionnaire (CGQ) results from 2005 to 2014 indicate that students believe “best suited to my personality” ranks among the top two on the list of influencing factors, the other being “personal interest.”^29^ These findings suggest that personality is an important consideration in specialty choice, and this study provides the first national data on the personalities of Canadian medical students. Given the aforementioned and that Canadian medical schools must provide guidance to students on their selection of electives and choice of residency,^30^ personality testing could become an important tool in the medical career decision-making process.

The principal investigator is currently examining how personality of medical students relates to specialty choice in Canada. It may be that students who demonstrate different characteristics on the KTS-II are attracted to certain specialties. In future, these types of studies might be expanded to determine the relationship of personality to the practice activities of doctors with respect to their roles in patient care, academia, scholarship and advocacy.

### Conclusion

This study indicates that the distribution of personalities in the Canadian medical student population is different from that of the general Canadian population. In addition, the distribution of personalities observed in each participating school shows the same directional trend as the weighted national average for medical schools.

## Footnotes

* Throughout the years of participation by Sherbrooke the administrator gave students the opportunity to participate in the study by approaching the first year class as a whole. Members of the class were never offered another opportunity to participate outside of this one-time whole-group process.

† Distribution of estimated Canadian general population (N ≈ 4,000,000) versus Canadian medical students measured in study (N = 3.435) differs significantly [X^2^=276.36, df=3, p<0.000; Cramer’s V=0.008, p< 0.000].

‡ Keirsey estimates the Canadian population as follows: Artisans 20-25%; Guardians 50-55%; Idealists 18-22%; Rationals 4-6%. These estimates are based on their “…observations of a sample size of more than 4 million individuals from a wide range of industry backgrounds throughout Canada.”^22^ The mid-points of the values provided through the Keirsey Assessments were used for the statistical analysis of the differences in the distributions.
